# Pathways of Climate Change Impact on Agroforestry, Food Consumption Pattern, and Dietary Diversity Among Indigenous Subsistence Farmers of Sauria Paharia Tribal Community of India: A Mixed Methods Study

**DOI:** 10.3389/fsufs.2021.667297

**Published:** 2021-06-17

**Authors:** Suparna Ghosh-Jerath, Ridhima Kapoor, Upasona Ghosh, Archna Singh, Shauna Downs, Jessica Fanzo

**Affiliations:** 1 Indian Institute of Public Health-Delhi, Public Health Foundation of India, Gurgaon, India; 2 Indian Institute of Public Health-Bhubaneshwar, Public Health Foundation of India, Bhubaneswar, India; 3 Department of Biochemistry, All India Institute of Medical Sciences, New Delhi, India; 4 Department of Urban-Global Public Health, Rutgers School of Public Health, New Brunswick, NJ, United States; 5 Berman Institute of Bioethics, Nitze School of Advanced International Studies (SAIS) and Bloomberg School of Public Health, Johns Hopkins University, Washington, DC, United States

**Keywords:** climate change, indigenous food systems, food consumption patterns, agroforestry, subsistence farmers, tribal community, smallholder farmers

## Abstract

Climate change poses severe threats to the social, cultural, and economic integrity of indigenous smallholder subsistence farmers, who are intricately linked with their natural ecosystems. Sauria Paharia, a vulnerable indigenous community of Jharkhand, India, are smallholder farmers facing food and nutrition insecurity and have limited resources to cope with climate change. Eighteen villages of Godda district of Jharkhand inhabited by Sauria Paharia community were randomly selected to conduct a mixed methods study. In 11 out of 18 study villages, we conducted focus group discussions (FGDs) to examine the perception of this indigenous community regarding climate change and its impact on agroforestry and dietary diversity. In all 18 villages, household and agricultural surveys were conducted to derive quantitative estimates of household food consumption patterns and agroforestry diversity, which were triangulated with the qualitative data collected through the FGDs. The FGD data revealed that the community attributed local climatic variability in the form of low and erratic rainfall with long dry spells, to reduced crop productivity, diversity and food availability from forests and waterbodies. Declining agroforestry-produce and diversity were reported to cause reduced household income and shifts from subsistence agricultural economy to migratory unskilled wage laboring leading to household food insecurity. These perceptions were supported by quantitative estimates of habitual food consumption patterns which revealed a predominance of cereals over other food items and low agroforestry diversity (Food Accessed Diversity Index of 0.21 ± 0.15). The adaptation strategies to cope with climate variability included use of climate-resilient indigenous crop varieties for farming, seed conservation and access to indigenous forest foods and weeds for consumption during adverse situations and lean periods. There were mixed views on cultivation of hybrid crops as an adaptation strategy which could impact the sustained utilization of indigenous food systems. Promoting sustainable adaptation strategies, with adequate knowledge and technology, have the potential to improve farm resilience, income, household food security and dietary diversity in this population.

## Introduction

Climate variability and change influence ecosystems, food security, health, and other domains fundamental to human existence and well-being (Costello et al., 2009; International Labour Organization, 2017). Indigenous people, who are the earliest inhabitants of a geographical region, share historical, cultural and physical connections with the environment and manage about 28 percent of the earth’s land surface (International Labour Organization, 2017). Rapid shifts in climate patterns have marginalized the livelihoods of already vulnerable indigenous populations globally, many of whom are predominantly smallholder subsistence farmers^1^ relying on rain-fed agriculture. Their geographical location in environmentally fragile regions (such as hills, forests, deserts, and floodplains), expose them to temperature and rainfall variability and a variety of climate risks, (Morton, 2007; Walpole et al., 2013) threaten their delicately balanced subsistence cycle, increase the likelihood of poor crop yields, and reduce their accessibility to culturally valued resources (Morton, 2007; Arbuckle et al., 2013).

The indigenous tribal communities of India, recognized by the government as “Scheduled tribes” (STs), are characterized by traditional belief systems, financial insecurity, and poor health outcomes (Tribal Health in India: Executive Summary Recommendations, 2018). Jharkhand, a tribal-dominated state in the eastern part of India, is experiencing the impacts of climate variability and change (Wadood and Kumari, 2009; Government of Jharkhand, 2014; Kumar et al., 2016; Ahmad et al., 2018; Tirkey et al., 2018), characterized by frequent occurrences of long dry spells, monsoon variability and extreme heat. Analysis of climatic trends in the state reveals a sharp decline in annual rainfall levels in several districts, with increased frequency and severity of extreme weather events like heat wave during summers, pre-monsoon hail storms, and extreme frost/cold wave in winters (Government of Jharkhand, 2014). Almost all districts are affected by drought; many regions experience forest fires and lightning, with sudden occurrences of heavy rainfall (Minj, 2013; Ahmad et al., 2018). This climate variability and resultant changes impact impoverished tribal communities in Jharkhand, whose livelihoods are dependent on subsistence farming and forestry that are an integral part of their indigenous food systems (Misra et al., 2008; Bhattacharjee et al., 2009; Ghosh-Jerath et al., 2020a, 2021). Further, environmental destruction due to deforestation and land degradation results in diminished biodiversity and further affects the integrity and stability of the ecosystems adversely (Kayet et al., 2016). All these changes have a marked effect on the social, cultural, and economic integrity of the indigenous tribal communities who are intricately linked to these ecosystems (Kayet et al., 2016).

Particularly vulnerable tribal groups (PVTGs) of India, who are poor, marginalized communities characterized by a pre-agricultural system of existence, zero or negative population growth and extremely low levels of literacy compared to other tribal groups, could be the worst-affected communities from climate related outcomes (Minj, 2013). Sauria Paharia, one of the PVTGs residing on the far-flung hilltops of Jharkhand, have diminished social and financial capital; they practice small-scale shifting cultivation for their livelihood, which often results in abject poverty and poor health status (Maternal Health and Nutrition Report, 2014; Ghosh-Jerath et al., 2020a,b). Despite their biodiverse surroundings, they are malnourished and face extreme levels of nutritional deprivation (Ghosh-Jerath et al., 2020a,b). They have limited access to technical and financial resources and development assistance programs due to geographical inaccessibility, (Maternal Health and Nutrition Report, 2014; Jamwal, 2019) leading to increased vulnerability to climate shocks.

Although there is an increased interest at the level of policy-making to ensure food security among indigenous communities in the face of climate variability (Vermeulen et al., 2012; Climate-smart agriculture for food security, 2014), a lack of information regarding the experiences of the community and their coping strategies hinders adaptation efforts. This gap in knowledge is particularly significant for the marginalized groups like PVTGs, who perhaps lack entitlements to participate in any policy process. Few studies from Africa (Mapfumo et al., 2016; Ayanlade et al., 2017; Zamasiya et al., 2017; Aniah et al., 2019) and Central America (Roco et al., 2015; de Sousa et al., 2018) have explored the responses and resilience of the indigenous smallholder farmers to climate change. These studies have reported high levels of climate vulnerability among the indigenous farmer communities, with specific impacts on their food sources and ecosystem services. However, limited literature exists on perspectives of Indian smallholder farmers belonging to indigenous tribal communities (Minj, 2013; Sharma, 2019). Climate variability and change threatens farmers’ food security and well-being in many tribal areas of Jharkhand (Minj, 2013; Sharma, 2019) and there is a need to have focused research on its impact on their food systems. This paper, hence, attempts to investigate perceptions about climatic variability, its influence on farming systems, food security, and local adaptation strategies among vulnerable Sauria Paharia tribes of Jharkhand, India. These findings are then discussed in the light of quantitative estimates of agroforestry diversity and household (HH) food consumption patterns in the community to triangulate multiple knowledge frames regarding climate variability impacts. The evidence base generated from this study will supplement the existing data on perceptions of indigenous smallholder farmers from different parts of the world, and may be helpful to understand their vulnerability and resiliency to climate variability in the Indian context.

## Materials and Methods

### General Characteristics of the Study Area

The study was conducted in Godda district of Jharkhand, a part of Santhal Parganas division of Jharkhand. The region is formed of undulating uplands, long ridges and depressions and is replete with scattered hillocks covered with forests. The total district area is 2110.4 km^2^, of which 35.2% is cultivable land and 11.2% is covered with forests (Godda District Administration, Jharkhand | India). The total scheduled tribe population is 279,208, which constitutes 21.26% of the total district’s population (Census, 2011). Godda district has a tropical climate, with average summer temperatures hovering around 41 degrees Celsius, winter temperatures around 28 degrees Celsius and average annual rainfall of 1094.9 mm (Godda District Administration, Jharkhand | India).

**Figure 1:**
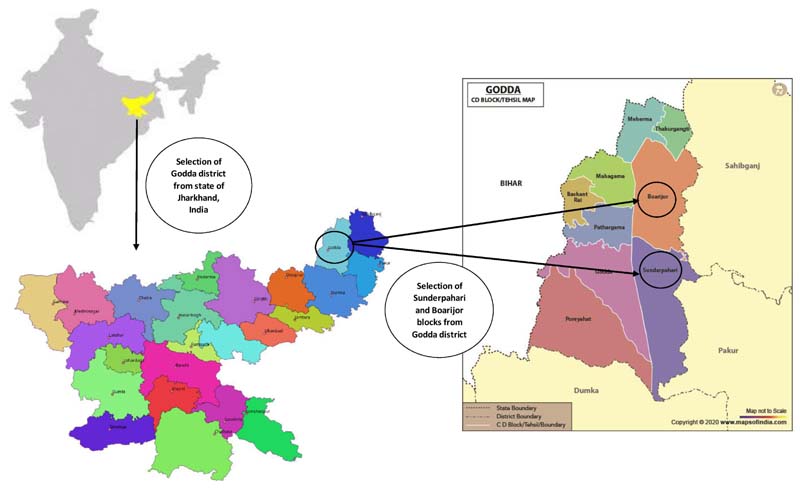
Study landscape in Godda district of Jharkhand, India.

Our study was focused in the geographically diverse Sunderpahari and Boarijor blocks of Godda district (Figure 1), with a high concentration of Sauria Paharia population (13,688) (Census, 2011). These two blocks were purposively selected to explore the impact of climatic variation in two geographically distant areas inhabited by the community. Sunderpahari has a total area of 433.4 km^2^ and spreads across hilly terrain with nearly 50% of the land covered with forests. It has a total scheduled tribe population of 50,133, divided across 221 villages. Boarijor has a total area of 388.04 km^2^ and is situated along both hilly regions and plains. The total scheduled tribe population of Boarijor is 76,935, spread across 309 villages.

### Study Design and Sampling

**Figure 2:**
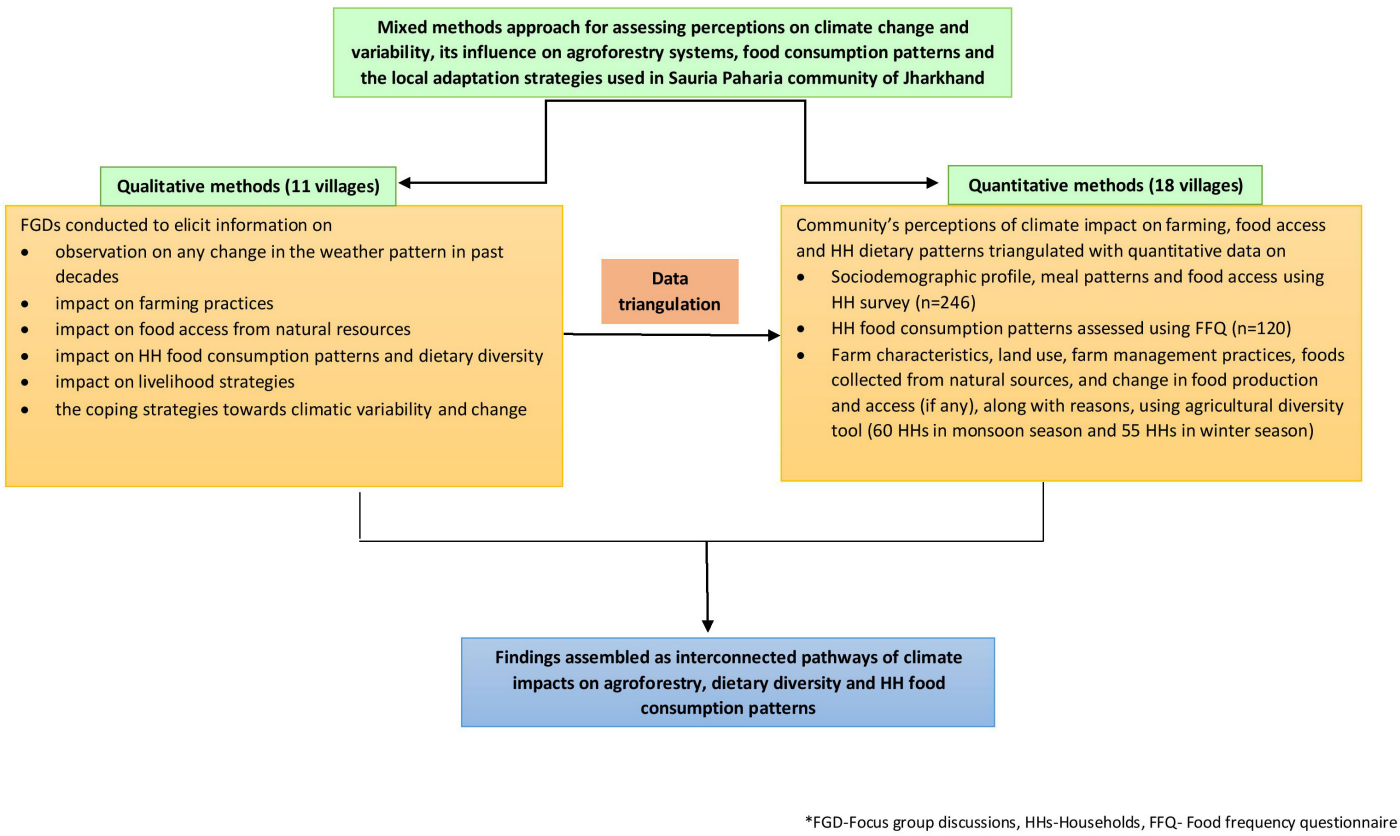
Methodological approach used in the present study.

This piece of research is part of a larger study which has used a mixed-methods approach for documenting the contribution of indigenous foods to nutrient intake and dietary diversity in tribal women and children of Jharkhand (Ghosh-Jerath et al., 2019). Figure 2 represents the detailed methodological approach followed in the present study.

A two-stage cluster sampling design was followed to select the villages and the HHs from the purposively selected blocks of Sunderpahari and Boarijor. In the first stage of cluster sampling, nine villages were randomly selected from each block (total 18 villages) using probability proportional to size sampling. Among these 18 villages, in March 2018, qualitative information was collected through focus group discussions (FGDs) from 11 villages (1 FGD per village) (6 villages of Sunderpahari and 5 villages of Boarijor) till the point of theoretical saturation. In the second stage of cluster sampling, all 18 selected villages were visited in June, 2018 and a house-listing exercise for all Sauria Paharia HHs was performed to construct the sampling frame of eligible HHs. The eligibility was based on the overall objective of the larger study and presence of at least 1 non-pregnant woman in the reproductive age group (15–49 years) and 1 child (6–54 months) in the HH. The eligible HHs were revisited in August 2018 for a detailed quantitative survey on HH level sociodemographic profile, food consumption patterns as well as HH access to different food sources.

Based on the overall objective of the study which explored the contribution of indigenous foods to dietary diversity and micronutrient intake, the requisite sample size (for quantitative survey) was calculated based on the difference in mean dietary intake of iron of 4 mg/day (35% increase) with a standard deviation (SD) of 7 mg/day between consumers and non-consumers of indigenous foods, reported in a previous study (Ghosh-Jerath et al., 2016) among women of Santhal tribes. Using the nMaster software (version 2.0) for sample size calculation, a sample size of 194 HHs was arrived at, with 80% power and a 5% level of significance, with a design effect of 2. Further details of the sample size calculation can be accessed elsewhere (Ghosh-Jerath et al., 2019).

### Qualitative Data Collection

For the qualitative inquiries, the empirical data were gathered through FGDs conducted to elicit the community’s perceptions of changes in the weather pattern and its impact on agriculture and coping strategies adopted. The respondents were selected using the snowball sampling technique (Naderifar et al., 2017) and represented different age groups to gather perceptions on past and present climatic conditions and their impacts (gender and age wise details are given in Table 1). The FGD guide was prepared by adapting questions from the tool “Climate Change and Food Security Vulnerability Assessment,” developed by Bioversity International and the Institute of Development Studies (Ulrichs et al., 2016). The FGDs were conducted in Hindi and the native Paharia dialect, with the help of trained local field workers fluent in the native dialect. All the FGDs were recorded using a voice recorder. Respondents were asked to discuss their views on a variety of topics such as their observation on any change in the weather pattern in past decades, its impact on farming practices, access to foods from natural resources, food consumption status, livelihoods, and coping strategies toward climatic variability. The principal investigator moderated the discussions with the help of a research assistant (for notes taking and recording) and a local field worker.

### Quantitative Data Collection

We conducted 246 HH surveys using a pretested structured questionnaire to elicit information on HH level sociodemographic profile, meal patterns and their access to different food sources. In addition to this, a detailed agricultural survey [adapted from Agricultural Questionnaire of the Third Integrated HH Survey, 2010-11, Malawi Government (National Statistical Office, 2011)] was administered on a conveniently selected sub-sample of 60 HHs in August 2018 (for a reference period of February to June 2018) and another 55 HHs in late January 2019 (for a reference period of July 2018 to January 2019) to capture detailed information on agricultural production and food access during the two cropping seasons (with sowing period during the monsoon and winter season, respectively). Using this tool, field investigators collected detailed data from the smallholder farmers of selected Sauria Paharia HHs on farm characteristics, land use, farm management practices, types of foods collected from different sources, and change in food production and access (if any), along with the reasons. The intent was to capture information on climatic variability (if any) as one of the possible reasons.

This information on HH food access and production (from HH survey and agricultural survey) was further utilized to construct an index called Food Accessed Diversity Index (FADI), which has been adapted from the crop diversity index (CDI) (Michler and Josephson, 2017). The FADI was created in order to provide an objective estimate of agroforestry diversity. For calculating FADI, the total number of foods grown, gathered or accessed and animals raised in a particular HH (n) were divided by the corresponding maximum possible number of foods grown, gathered, accessed and raised in a particular village (*N*). Foods accessed from the market were not included in this index. The FADI was expressed as

$$\text{FADI=}{\left(\frac{\text{n}}{\text{N}}\right)}^{\text{2}}$$

Lower values of FADI indicate lower diversity in production and access to foods and vice versa.

The frequency of consumption of different food items under various food groups at HH level was assessed using a food frequency questionnaire (FFQ). Food items were identified during FGDs conducted in the month of March and were extensively used to develop the FFQ that included both commonly consumed Indian food items as well as indigenous tribal foods from the region. We developed a 300-item FFQ and administered it on a conveniently selected sub-sample of 120 HHs during the monsoon season (August 2018). The questionnaire inquired about the frequency of consumption over the past 1 month without specification of portion size. Nine predefined frequency categories ranging from “never” to “2 or more times per day” were used.

All the questionnaires were piloted in the field prior to data collection. HH and agricultural surveys were administered using handheld tablets and CS pro software (Version 7.1), by a team of enumerators who underwent formal training. Paper forms were used for FFQ and administered by nutritionists and nutrition interns after due training. All surveys were conducted with either the HH head or family member in charge of the HH.

### Data Analysis

FGDs were recorded and transcribed verbatim from Paharia to Hindi followed by translation of Hindi transcripts to English. The data were coded using Atlas.ti version 8. The data were first open coded and subsequently combined into key themes using a thematic framework analysis, for identifying, analyzing and generating relevant themes within the data (Braun and Clarke, 2006). For quantitative data, descriptive statistics were used wherein continuous variables were reported as mean ± standard deviation (SD) and categorical ones were summarized using counts and percentages. Quantitative analysis was performed in Stata version 15.

### Ethics Approval

The study was conducted according to guidelines laid down in Declaration of Helsinki (The World Medical Association-WMA Declaration of Helsinki, and Ethical Principles for Medical Research Involving Human Subjects, 2018), and all procedures involving humans in this study were approved by the Institutional Ethics Committee at Indian Institute of Public Health-Delhi, Public Health Foundation of India and All India Institute of Medical Sciences, New Delhi. Administrative approvals from authorities at district level as well as cluster level consent from the village leader were obtained. Literate FGD participants provided written informed consent while third-party witnessed verbal consents were taken from those who were illiterate.

## Results

### Household Characteristics, Food Production, and Access of Sauria Paharia Community

**Figure 3:**
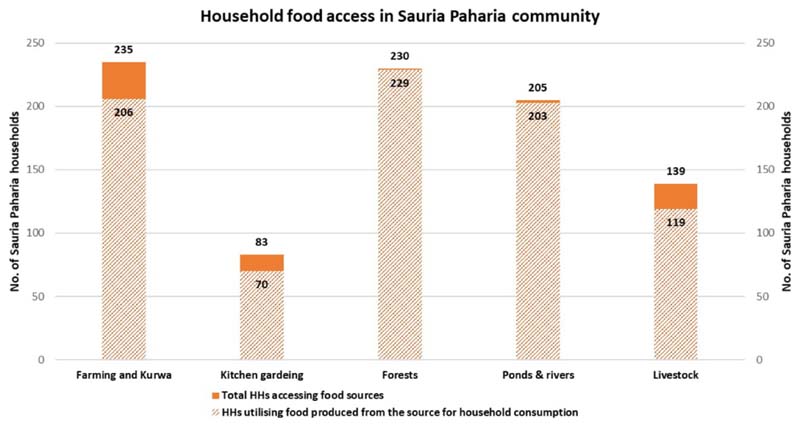
Household food access from natural sources in Sauria Paharia community (*n* = 246). *Kurwa refers to utilization of small patches of forest lands for slash and burn cultivation.

The HH survey revealed that Sauria Paharias are mainly smallholder subsistence farmers, who usually have poor living conditions in terms of housing, access to clean cooking fuel, electricity, transport facilities and poor literacy (Table 2). Most HHs (96%) practice settled agriculture and shifting cultivation/*Kurwa* farming (utilization of small patches of forest lands for slash and burn cultivation), while about a third of the HHs utilize their backyards as kitchen gardens (*Bari*). The majority of the HHs (60%) manage livestock like cows (mostly for draft power), raise goats, pigs, and do poultry farming; while almost all HHs reported accessing local forests, water bodies and surrounding areas for collecting indigenous varieties of foods for consumption (Figure 3). The community also accesses government food supplementation programs such as the Integrated Child Development Services and Mid-Day Meal Scheme [Integrated Child Development Services Scheme, 2009, Mid Day Meal Scheme Ministry of Education, Government of India] as well as national food security schemes [Public Distribution System (PDS), that provides staple food grains (wheat and rice) and other commodities such as sugar, salt etc. at subsidized prices to poor HHs] (The Public Distribution System, 2019).

### Climate Variability and Its Impacts on Agroforestry Systems and Dietary Diversity

**Figure 4:**
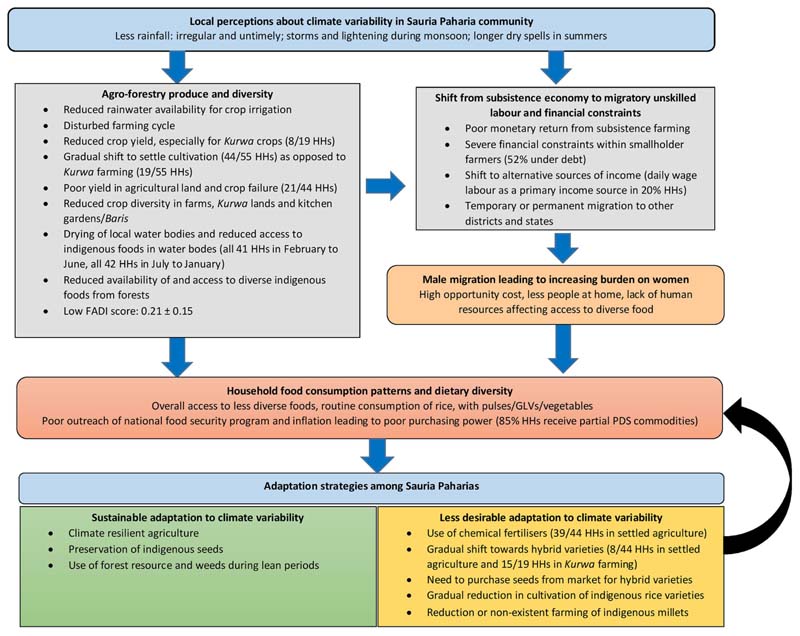
Interconnected pathways of climate change impact on agroforestry systems, food consumption patterns, and dietary diversity among Sauria Paharias, Jharkhand, India.

Based on the thematic analysis of the qualitative inquiries on community’s perceptions along with quantitative data from HH and agricultural surveys, we explored the interconnected pathways through which climate variability impacted the agricultural production, HH dietary diversity, and food consumption patterns. We also identified certain adaptive strategies employed by the community to cope with these changes. These perceived impacts and the adaptive strategies were then assembled as pathways of climate impact on agroforestry systems and dietary diversity (Figure 4). Each of the steps of this figure is a compilation of the different themes that emerged from our analysis. These were then triangulated with the findings from quantitative surveys. The steps of this pathway include: (i) community’s perceptions regarding climate variability observed in the region, (ii) the proximal impact of climate variability on agroforestry produce and diversity (iii) its distal impact leading to shift from subsistence economy to migratory unskilled labor with financial constraints, which has resulted in (iv) male migration leading to increasing burden on women, and, how this has (iv) cumulatively impacted the HH food consumption patterns and dietary diversity; and lastly (v) the adaptive strategies identified by the community to cope with climate variability and its implications. These themes are described and detailed as follows:

#### a) Community’s Perception on Climate Variability in the Region

During the FGDs, the respondents reported changes in the local weather pattern which manifested as declining rainfall trends over the past two decades as well as irregular rainfall followed by long dry spells. Some also reported witnessing strong wind and thunderstorms during the monsoon season. As shared by one respondent: “*Water which used to fall (in rain), was sufficient, but now it doesn’t rain that much, and even if it rains, it rains in the wrong season, it comes with lightning and in fact the rain is less and lightning is more*” (Respondent number 2, female, study village two, Sunderpahari block), while another respondent stated: “*There is very less rainfall now, because of that there is drought in the village*” (Respondent number 1, male, study village two, Boarijor Block).

#### b) Proximal Impact of Climate Variability on Agroforestry Produce and Diversity

According to FGD respondents, variations in local weather conditions are impacting their agricultural practices and kitchen garden produce. Water scarcity has reportedly become a major issue in the region due to long dry spells. Residing in isolated hilly regions with limited access to man-made water resources, the community mainly relies on natural sources like rain, mountain springs and wells for water. However, owing to the climatic variability, the respondents reported that most natural water resources usually dry up resulting in severe water scarcity, especially during the summers. The respondents further reported an inordinate reliance on rain-fed agriculture due to financial constraints preventing access to mechanized farm irrigation systems. Hence, they felt that anomalies in usual rainfall patterns have led to reduced productivity in their farms and kitchen gardens, along with crop failure in forest lands. They also highlighted the impact of untimely and delayed monsoon on their cropping cycle and yield due to disruption of sowing and germination cycles. One of the respondents shared, “*In the earlier times we used to have timely rain, so the crops would also grow on time. Now what happens is, at times it rains, at times its sunny”* (Respondent number 3, male, study village six, Sunderpahari block).

Reduced agricultural yields were reported for indigenous varieties of cow pea (*Ghangra*), pearl millet (*Shishua*), and red gram (*Rehad*) resulting in their reduced cultivation. Respondents from some villages (in Sunderpahari block) reported that *Kurwa* farming, which was a common practice previously, has now been replaced with farming in plains, which has affected the crop diversity. The current varieties of crops grown are reportedly restricted to paddy varieties only, although historically, a diverse range of indigenous crops like finger millet (*Mandua*) and little millet (*Gondli*) were grown. One respondent said: “*If there is rainfall, then proper farming is there. If we get any source of irrigation then we can plant tomato, ladyfinger, and others. Because of low rainfall, soil dries, there is no source of irrigation, that is why, we have one crop only and the entire year goes by like this*” (Respondent number 10, male, study village five, Boarijor Block).

**Figure 5:**
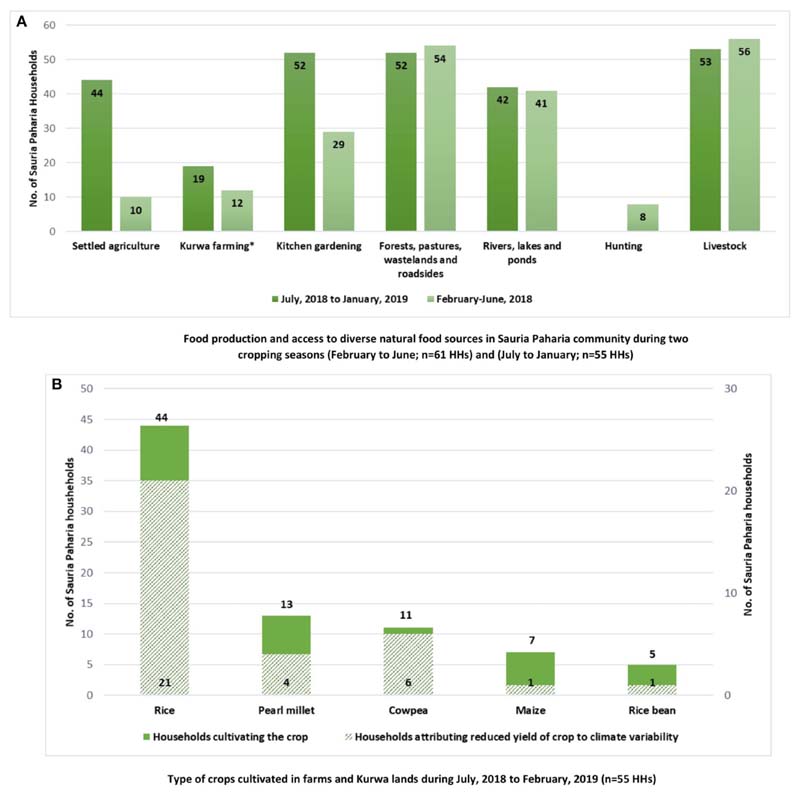
**(A,B)** Agroforestry production and diversity in Sauria Paharia community, Jharkhand, India. *Kurwa farming refers to utilization of small patches of forest lands for slash and burn cultivation.

Information from agricultural surveys revealed that most of the HHs mainly practice farming during July to January, with the local monsoon season falling between June and September. Among 55 HHs that were interviewed during the main cropping cycle, a total of 44 HHs reported practicing paddy cultivation on plain farmlands and 19 HHs practiced *Kurwa* farming around the months of November-January, wherein they cultivated crops like pearl millet, cowpea (*Ghangra*), maize (*Makai*), and rice bean (*Suthro*). Respondents experienced reduced yields of these crops and attributed it to insufficient or erratic patterns of rainfall in the region. Almost all HHs grew foods in kitchen gardens or *Baris*, although majority of the HHs grew two types of crops (usually maize and mustard leaves) while more than a third practiced mono-crop cultivation (Figures 5A,B).

During the FGDs, the community reported historical use of a wide variety of indigenous foods from forests and local water bodies. However, a decrease in the availability of these foods was reported in the present times, which was attributed to climate variability. At present, indigenous varieties of fruits, mushrooms and leafy vegetables are accessed from wild food environment, but their availability has reportedly reduced over the past two decades. Indigenous fishes, that used to be frequently consumed in the past, have now become difficult to access, due to drying of rivers, lakes, and ponds. The community reported that water scarcity has resulted in considerable hardships to fulfill basic necessities of life like potable water for drinking and cooking and water for other purposes like bathing, cleaning, etc. One of the respondents commented: “*There is no water in the river also, which used to be there in olden days. For 10–15 years, there has been a shortage of water here. Earlier there were small streams which had water all the year round. These days everything has dried up. The amount of produce (in forest) has reduced as compared to earlier days, because of this change in weather*” (Respondent number 3, female, study village four, Boarijor Block).

The data collected in the agricultural surveys also revealed that the local forests and open spaces (pastures, wastelands, roadsides) were accessed for foods only once a week by a large majority of the HHs in the two cropping seasons (31/52 HHs in July to January and 29/54 HHs in February to June). Apart from infrequent access, only a limited number of foods (one to two varieties) were reportedly gathered by a large majority (46/52 HH in July to January; 29/54 HHs February to June). Declining availability of wild foods was reported by a few HHs (12/54 HHs in February to June) which was attributed to spoilage by pests/insects (5/12 HHs). Hunting of animals was very rarely practiced in both the seasons. During both the agricultural survey periods, all HHs reported reduced varieties of fishes in ponds, rivers and lakes, along with drying up of water sources. They attributed these toward local climate variability like reduced frequency and intensity of rainfall. In accordance with the lesser variety of foods accessed/produced despite access to diverse natural food sources like agricultural land, *Kurwa, Bari*, forest, and open access areas, the mean Food Accessed Diversity Index (FADI) for this community was also observed to be very low (Table 2).

#### c) Shift From Subsistence Economy to Migratory Unskilled Wage Laboring and Financial Constraints

According to the FGD respondents, the impact of climate variability on local agricultural practices has resulted in several social and economic outcomes. The diminishing agricultural yield has resulted in reduced availability of food for consumption and has impacted the monetary benefits gained by the HHs through selling of surplus produce in the local market or to middlemen. This has led to severe financial constraints leading to hardships and difficulty in managing basic HH expenses. For instance, a respondent shared: “*We usually sell suthri (an indigenous pulse) for our household income. But, when there is need for more money, we sell all crops produced by us during the year. We do not keep anything for household consumption. Though this should not be practiced but we feel helpless during hard times and are forced to sell these*” (Respondent number 5, female, study village four, Sunderpahari Block). During the HH survey, about one-third of the respondents reported facing an outstanding debt which was attributed to reduced income from agriculture (52%). The qualitative enquires revealed that the community has started adopting alternative sources of livelihood and is gradually shifting to agricultural and daily wage labor, construction work, and firewood collection in order to address this HH financial hardship. These findings are further corroborated with the HH survey data, which also revealed that wage laboring is the primary source of income in 20% HHs and secondary source of income in 28% HHs.

#### d) Male Migration Leading to Increasing Burden on Women

During the FGDs, the respondents informed that usually, the male head of the HH or other adult male member and sometimes the entire family temporarily migrates in search of work to other districts of Jharkhand or states like Maharashtra, Gujarat, Haryana, West Bengal, and the National Capital Territory of Delhi. In the absence of male members of the family, the adult women usually take charge of the farming activities in addition to carrying out usual HH chores, managing livestock and rearing of children. The crop yield and forest sojourns for collection of foods and items like firewood etc. are also affected as women can devote less time for these activities. In other words, the opportunity cost of accessing forest items along with the management of farms has increased; thus, negatively impacting the diversity of cultivated and wild food sources. One respondent noted: “*Effect of migration on our farming exists. At night, we need to take care of our farms. In the night, wild pigs come and eat up all the grains that are there. In the daytime, monkeys come and eat our crops. The men migrate, women are already doing their household chores, as a result we face loss of crops*” (Respondent number 1, female, study village one, Boarijor Block).

#### e) Cumulative Impact on Household Food Consumption Pattern and Dietary Diversity

During the qualitative enquiries, it was reiterated that climate variability, apart from affecting agroforestry and livelihood patterns, have further led to poor availability of and accessibility to diverse foods including indigenous varieties. This has indirectly affected the HH food consumption patterns and dietary diversity of the community. The community expressed that owing to a decline in crop productivity, suboptimal use of kitchen gardens and forest degradation, the farm and forest produce are insufficient and less diverse to meet the HH’s needs. For instance, a respondent shared: “*Climate change is affecting our farm produce. If the farm produce is less, then less food is available for household consumption*” (Respondent number 5, female, study village two, Sunderpahari Block).

A significant dependence on food commodities like rice, wheat, sugar etc. distributed through PDS at subsidized prices, was reported. However, owing to supply chain issues and erratic outreach of the program, it was considered inadequate to support the food security needs. One respondent commented: “*Though we get rice (distributed under PDS), it’s too less, we eat that for a few days, but for how long will it suffice. We are feeding the family, if 30 kg has been given, for how long will it suffice. In 10–15 days, it will be finished. We get ration only once in 2 months, sometimes in 6 months*” (Respondent number 2, male, study village four, Boarijor Block). Table 2 on the HH survey data also shows that large majority of HHs (85%) receive only partial amounts and kinds of food commodities through PDS.

During the FGDs, the respondents indicated that local markets are routinely accessed to meet food requirements for the families. However, owing to general inflation in food prices, many food items (such as rice) have become expensive, thereby lowering their purchasing capacity. One respondent claimed: “*The rice which we used to eat at Rs. 14 (0.19 USD) per kg, are now sold at a rate of Rs. 25–26 (0.33–0.35 USD) per kg*” (Respondent number 1, male, study village four, Boarijor Block). All these challenges have resulted in scarcity of food and several HHs are unable to eat two square meals a day. A respondent commented: “*We do not have enough crop, enough land, there is nothing to eat all the year round in our house. There are only 1–2 people who can eat all the year round, there is no assurance that if we can arrange for food today, we will be able to arrange food for tomorrow also. There is nothing to feed our children throughout the year. Kota (PDS) rice is given by government so somehow we are managing otherwise the rice from market has become very expensive*” (Respondent number 2, male, study village five, Sunderpahari Block).

The qualitative inquiries further revealed that the diets historically consumed by the community (about two decades ago) usually comprised of indigenous rice, vegetables, and roots and tubers, which were flavorsome and provided nourishment and satiety. However, presently the community depends on a predominantly cereal based diet, consisting of rice with small amounts of pulses, roots, and tubers (potatoes) and/or green leafy vegetables. Respondents felt that this shift in dietary patterns has drastically compromised the nutritional quality of their meals. They further reiterated the impact of changing climate on diminishing availability of indigenous foods presently, which were abundantly available historically. As shared by a respondent: “*The indigenous varieties are tasty and provide strength. If we consume hybrid, we digest it rapidly and feel hungry again. If we eat indigenous food, we feel full for a longer duration*” (Respondent number 7, female, study village four, Sunderpahari Block).

The diet diversity assessed by exploring the food consumption data at the HH level using the FFQ [for the monsoon season, (*n* = 120)] also revealed routine consumption of cereals (mostly rice), other vegetables and roots and tubers (Table 3). Once a week consumption was observed for pulses, flesh foods and leafy vegetables in only half of the HHs. Seasonal fruits like mango and dates were reportedly consumed daily in about one-fourth of HHs. Milk and milk products were rarely consumed, with only 17% of HHs reporting once or twice a month consumption of cow or buffalo milk. Routine consumption of market-procured packaged and freshly prepared foods (like sweets, biscuits and savory snacks) were reported in about 32% HHs, while one-fourth HHs reported once or twice a week consumption.

#### f) Adaptation Strategies of the Community Toward Climatic Variability and Change

The community highlighted that climate variability, including erratic rains and dry spells, has significantly affected their nutritional, social and economic well-being. In view of these changes, they shared several strategies that they have adopted to cope with the changing climatic conditions. While some of these strategies may have beneficial environmental impact, certain adaptation strategies could pose threats to the historically evidenced sustainable methods of farming and food collection.

There are three main ways in which smallholder farmers reported adapting to climate variability: (1) utilization of traditional ecological knowledge for retaining use of climate resilient (drought tolerant) and less resource intensive indigenous varieties of crops like rice (*Bhadai Dhan* and *Swarna Dhan*), pearl millet, horse gram (*Kulthi*) and cow pea. In addition to this, indigenous seeds are also preserved using traditional methods like sun-drying the seeds and wrapping them in medicinal indigenous Sinduar leaves (*Chilo Ghasi*) to store for use in the next sowing cycle; (2) reliance on forests during lean periods to access indigenous roots and tubers (e.g., *Nappe*, Sweet potato/*Shakarkand*), wild fruits (Marking nut/Kero, *Kend*, and *Dumari*), green leafy vegetables (Koinar leaves) that grow in adverse climatic conditions; and (3) incorporation of modern farming techniques such as the use of hybrid seed varieties and chemical fertilizer for better crop productivity.

The qualitative enquiries further revealed that the practice of adopting modern approaches to farming has been met with mixed views from the community owing to its financial implications as well as the issues around organoleptic quality (taste) of the crops produced. The respondents stated that the indigenous rice varieties (such as *Bismunia* and *Dumarkani*), which were consumed by the older generations for their flavorsome taste, have now become almost non-existent or extinct. The respondents also shared their views on the farming of traditional coarse millets, highlighting that their cultivation has drastically reduced due to poor yields in *Kurwa* lands and the community’s adaptive strategy of switching to paddy cultivation in plain agricultural lands. On further enquiries, the respondents stated that these practices have been reinforced by the agricultural policies and local agricultural extension organizations which have been promoting high yield hybrid varieties with a primary focus on better yield. Consequently, many indigenous varieties of rice (like *Bahiar Dhan and Lal Dhan*) and maize (*Potio Makai*) have now been replaced with their hybrid counterparts. Likewise, millets like (*Gundli* or little millet), earlier consumed ubiquitously, have presently become virtually extinct. All these adaptations have also led to financial implications (purchasing of hybrid seeds, fertilizers for cultivation), which is further pushing the community into a vicious cycle of food insecurity. Though the community values its traditional practices, it has forcibly adapted these practices from a sheer survival perspective. One respondent said: “*Lal dhan (indigenous variety of rice) used to be the major crop here. After cooking, its aroma spreads even outside the house. Six to seven years back, we used to sow only this paddy, but now it does not grow. Before harvesting, all the water dries up, so paddy does not grow well. That is why hybrid rice is sown. Whether water is there or not, the yield is good, but it is not tasty*” (Respondent number 5, male, study village one, Boarijor Block).

A similar shift toward modern farming techniques were reported in the agricultural survey. During the main cropping cycle, although most HHs (36/44) reported cultivating indigenous rice varieties (i.e., *Swarna* rice), a few HHs (8/44) reported utilizing hybrid paddy seeds. In case of *Kurwa* farming, all HHs reported growing hybrid maize and cow pea, while in case of crops like pearl millet and rice bean, only indigenous seeds were used. Use of chemical fertilizers was reported by most HHs (39/44) in settled agriculture, and for *Kurwa* farming, only one HH reported the use of fertilizers while the rest (18 HHs) used neither organic manure nor fertilizers.

## Discussion

This paper examines the perceived impacts of climate variability among smallholder subsistence farmers of the Sauria Paharia community. These findings are further triangulated with quantitative estimates on agricultural diversity and food consumption patterns at HH level. Based on our qualitative inquiries, it was ascertained that climate variability has led to water scarcity in the region, affecting agroforestry production and diversity. This has a cascading effect and amplifies uncertainties around livelihoods, financial and manpower constraints, and impacts HH food consumption pattern and dietary diversity. Coping strategies to address these interlinked uncertainties included the retention of climate resilient indigenous varieties of crops, use of foods from natural vegetation and forests in lean periods and adoption of modern farming practices and hybrid varieties of crops. The quantitative data also provided objective estimates of diminished agroforestry and dietary diversity and attributing factors related to climate variability.

The community was found to be aware of climate variability and principally perceived it as changing rainfall patterns (both frequency and intensity) accompanied by long dry spells. Similar perceptions on climate variability have been recorded among smallholder famers across Jharkhand and other Indian states (Kelkar et al., 2008; Barua et al., 2014; Varadan and Kumar, 2014; Banerjee, 2015; Shukla et al., 2016; Ghosh-Jerath et al., 2021). The perceptions in this study coincide with the scientific data reported by the Indian Meteorological Department (“Indian Meteorological Department”) and other literature suggesting an overall increasing trend in summer temperatures in Jharkhand during the last 20 years (Tirkey et al., 2018). The rainfall trend in Jharkhand also appears erratic in last 20 years with a 26–270 mm decrease in the northern region where Godda is located (Tirkey et al., 2018). Several anthropogenic elements like land use changes, deforestation and environmental degradation have been documented as possible factors contributing toward the patterns observed, especially in the state’s northwestern districts like Godda (Riebsame et al., 1994; Zhou et al., 2004; Rawat and Kumar, 2015).

The community recognized that the local climate variability as long dry spells and erratic rains has affected the farm productivity and diversity (due to water stressed environment). These changes have also affected the availability of indigenous foods from natural vegetation, forests, and waterbodies in the region. The quantitative surveys also revealed similar climatic factors like erratic rainfall patterns contributing toward diminished agricultural production. The literature indicates that increasing summer temperatures may have led to water scarcity in the region (Tirkey et al., 2018), and erratic rainfall has impacted agricultural yields, especially for paddy cultivation. According to Jharkhand Action plan on climate change (2014), high rainfall (untimely rain) in rice flowering stage has caused reduced rice yield (Lal, 2001). There is also a gradual shift from rich and diverse multi-cropping patterns to intense mono-cropping (paddy cultivation) on flat lands. The agricultural survey revealed an increasing trend toward mono-cropping in the study villages, with preferential cultivation of paddy over indigenous crops like cowpea, pearl millet and rice bean. This trend is in fact being followed in the entire state of Jharkhand now. As per the recent state survey report, the total area under paddy cultivation has increased at an annual rate of 4.5 per cent, while the cultivation of pulses (red gram and black gram) have decreased at the annual rate of 0.8 per cent and 13.3 per cent, respectively (Jharkhand Economic Survey 2018-19, 2019). All these may impact the overall dietary diversity of the smallholder farmers who substantially rely on their home-grown foods.

Our findings from both qualitative and quantitative surveys have highlighted the climate impacts on the agroforestry systems, which in turn is negatively influencing the HH food availability and access. This includes foods procured from cultivated and wild sources as well as foods purchased from local markets (through indirect impact on purchasing capacity). A poor FADI score was also reported, which indicates poor diversity in terms of HH food access and production. The low FADI scores were further reflected in the consumption patterns at the HH level, with dominance of cereals (particularly rice) in a typical HH diet. The community also reported a shift from a historically diverse diet to a monotonous diet at present. Another study among Sauria Paharias revealed decreased HH access to foods collected from forests, farms and water bodies, which has resulted in reduced consumption of indigenous varieties of maize, millets, leafy vegetables and flesh foods (Mishra, 2017). Studies have also documented widespread prevalence of food insecurity among other tribal communities of Jharkhand as well as across different tribal settlements in the country (Tagade, 2012; Ghosh-Jerath et al., 2016; Chyne et al., 2017; Yasmin et al., 2018; Sharma, 2019).

Owing to diminished yield and reduced farm generated income, a shift from a subsistence economy to migratory unskilled wage laboring was reported. Migration among the smallholder farmers and tribal communities have been documented from other parts of the state as well as other states of India (Mungreiphy and Kapoor, 2010; Vermeulen et al., 2012; Das and Das, 2014; Bharali et al., 2017; Chandra and Paswan, 2020). A comparison between Census data from 2001 and 2011 shows that the proportion of ethnic groups engaged in agriculture has reduced by more than 10%, while the proportion of tribal wage laborers has increased by 9% (Census, 2011). Our study findings showed daily wage laboring as a significant income source in about one-fourth HHs and as a secondary income source in one-fifth HHs. This shift in occupational patterns may lead to a change in the economic status and social dynamics within a HH. With a rapid nutrition transition underway, when, even remote rural communities are not spared from its impact, an increased reliance on the market for sourcing food and other basic requirements of day to day life could lead to a shift in the composition of family diets. According to the state report on Jharkhand, climate impacts have diminished the agricultural and forest productivity of many smallholder farm communities, that has further impacted their HH food security, resulting in dietary deficiencies of fruits (69%), milk (43%), meat (35%), and food grains (14%). As a coping strategy, many vulnerable and poor communities in Jharkhand have shifted their dependence to market foods to meet their consumption needs (Government of Jharkhand, 2014). Quantitative estimates from our study further revealed routine consumption of ready-to-eat market foods, which are mostly cheap sources of energy and are rich in fat, sugar and salt. Some studies have also reported increasing consumption of processed foods among tribal communities, due to their changing occupational patterns (Mungreiphy and Kapoor, 2010; Barbhuiya and Das, 2014; Das and Das, 2014; Bharali et al., 2017; Ghosh-Jerath et al., 2021). The challenge of managing farms and accessing forests for wild foods when male members migrated to towns and cities was highlighted in the study. The migration of an adult member of the family can thus add to the opportunity cost of accessing diverse sources of food from wild and cultivated food environment (Ghosh-Jerath et al., 2021).

Certain coping strategies reported include cultivation of hybrid varieties of crops using chemical fertilizers and promotion of non-indigenous varieties of crops for better land productivity. However, existing literature claims that hybrid crops are extremely climate sensitive while overuse of chemical fertilizers further reduces the soil fertility. Hence, over emphasis on yield over nutritional qualities may pose a threat to the self-sustainable and environment friendly attributes of the indigenous food systems and lead to several socio-ecological implications such as biodiversity loss, soil degradation, increased vulnerability to climate variability and erosion of traditional ecological knowledge (Eliazer Nelson et al., 2019).

However, we also found some effective adaptation strategies that the community used to foster climate resilience in food production and access, while preserving their natural biodiversity. These include use of climate resilient indigenous varieties of crops, seed conservation and utilization of transgenerational traditional ecological knowledge for accessing indigenous varieties of forest foods and weeds for consumption during adverse situations. This preference toward revival and cultivation of indigenous crops has a strong sustainability component, provided the community is supported and empowered with knowledge and technology to support the production and consumption of traditional foods. For instance, with the help of local NGOs and organizations, smallholder farmers in tribal regions of states of Maharashtra, Tamil Nadu, Odisha, Karnataka and Uttarakhand have successfully revived the cultivation of indigenous crops with special focus on minor millets, thus improving their income and strengthening their food and nutrition security (Fanzo et al., 2013; Bose, 2017). Similarly, agrarian tribal communities from Madhya Pradesh and Chhattisgarh are returning to their traditional crop diversification methods which are cost-effective and give an assured yield in both low and excess rainfall conditions (Mahapatra, 2018; Mahapastra, 2020).

### Study Limitations

Owing to the detailed nature of the agricultural survey and FFQ and limited time available with the respondents due to ongoing sowing season, the tools were administered on a sub sample of the study population. Although the study team had field staff speaking the local language, language and cultural barriers may have influenced some responses.

## Conclusion

Assessment of perceptions and experiences of climate variability among Sauria Paharias and the quantitative surveys highlight the important issues concerning their food systems and livelihood patterns and suggest an urgent need to manage traditional natural resources and reduce the impacts of climate uncertainties. A significant continuing decrease in availability of locally sourced indigenous foods and erosion of indigenous seed varieties appears to be underway with serious implications on agroforestry, dietary diversity and diet quality. Our data also provides important insights into the sustainable adaptation practices used by the community, that have the potential to improve agricultural outcomes and food security by improving farm resilience, income and diet diversity, preserving the natural ecosystems and providing genetic resources for future climate adaptations. These sustainable adaptation strategies need to be supported by policies, programs and behavior change communication interventions that build on the strengths of indigenous food systems and promote climate-smart and genetically diverse agriculture for improved food, nutrition, and livelihood security of this population. While indigenous smallholder farmers covered in the present study are vulnerable to climate variability, they may also provide critical information that can be utilized to plan interventions for making current agricultural systems sustainable and climate resilient.

## Figures and Tables

**Table 1:** Characteristics of FGD respondents in villages of Sunderpahari and Boarijor blocks of Godda district, Jharkhand, India.

Block	Study village	Respondent group size	Men	Women	Elderly	Young middle- adult
Block 1	Village 1 Tasaria	9	5	4	✓	✓
Sunderpahari	Village 2 Kusumghati	7	1	6		✓
	Village 3 Paharpur	10	1	9	✓	✓
	Village 4 Chewo	8	4	4	✓	✓
	Village 5 Longodih	9	3	6		✓
	Village 6 Nadgoda	4	1	3		✓
Block 2	Village 1 Rajabhita	11	4	7	✓	✓
Boarijor	Village 2 Kusumghati	15	11	4	✓	✓
	Village 3 Lutibahiar	10	2	8		✓
	Village 4 Bara-amra	8	4	4		✓
	Village 5 Kortica	9	4	5	✓	✓

**Table 2:** Household characteristics, food production and access of Sauria Paharia community in Jharkhand, India (*n* = 246).

Household characteristics	n (%)
**House type**	
*Kaccha* (mud/thatched roofs and walls)	190 (77.2)
*Semi-Pakka* (semi-cemented roofs and walls)	51 (20.7)
*Pakka* (cemented roofs and walls)	5 (2.1)
**Source of cooking fuel**	
Firewood/chips/grass/stems/straw/shrub	230 (93.5)
Kerosene	10 (4.3)
Others	6 (2.2)
**Source of drinking water**	
Tube well/Hand pump	177 (71.9)
Well	57 (23.2)
River/dam/spring/waterfall	10 (4.1)
Piped water/tank	2 (0.8)
**Source of light**	
Kerosene oil	152 (61.8)
Solar panels	51 (20.7)
Electricity	34 (13.8)
Biogas/Gobar gas	2 (0.9)
Others (other oils/candles)	3 (1.2)
None	4 (1.6)
Access to farm inputs (transport, farm, and irrigation equipment)*	
Bike/scooter	23 (9.3)
Bicycle	141 (57.3)
Bullock cart	2 (0.9)
Farm equipments (non-mechanized)	102 (41.5)
Farm equipments (mechanized)	2 (0.8)
Irrigation motor pump	6 (2.4)
**PDS Ration card**	
Yellow (AAY)	153 (62.2)
Red (BPL) 40	(16.3)
Don’t have	53 (21.5)
**PDS Utilization**	
Full entitled ration 29	(15.02)
Partial ration^	164 (84.9)
**Access to Anganwadi services by children**	
Everyday	90 (36.7)
3 or more than 3 times a week	42 (17.1)
< 3 times a week	52 (21.2)
Never 61	(24.7)
**Access to Mid-day meal services**	
Everyday	115 (46.9)
3 or more than 3 times a week	45 (18.4)
< 3 times a week	46 (18.8)
Never	13 (5.3)
Not Applicable	26 (10.6)
**Literacy level of HOH**	
No formal education	122 (49.7)
Less than primary (till 4th standard)	22 (8.9)
Primary but less than secondary (till 9th standard)	80 (32.5)
Secondary (10th standard) & above	22 (8.9)
**Occupation of HOH**	
Settled Agriculture/ Shifting cultivation	192 (78.1)
Daily wager (agriculture & non-agriculture)	37 (15.1)
Hunter/Gatherer	5 (2)
Craftsmen/artisans/ Service (Government and Private)/Self-employed	9 (3.6)
Unemployed	11 (1.2)
**Number of main meals consumed**	
1 meal	26 (10.6)
2 meals	153 (62.2)
3 meals	66 (26.8)
>3 meals	1 (0.4)
**Consumption of outside meals**	
Yes	136 (55.3)
No	110 (44.7)
**Number of times family eats outside meals**	
Everyday	12 (4.9)
More than 3 times a week	17 (6.9)
2–3 times a week	26 (10.6)
Once a week	14 (5.7)
Once in a fortnight	4 (1.6)
Once in a month	20 (8.1)
Sometimes	43 (17.5)
Type of outside meals consumed*	
Freshly prepared meals	126 (92.6)
Ready-to-eat meals	114 (83.8)
Convenience packaged foods	127 (92.6)
Non-perishable sweets	122 (89.7)
Perishable sweets	123 (90.4)
**FADI Score (Mean ± SD)**	0.21±0.15

* Multiple responses captured; percentages do not total 100%.

^ Either limited food commodities or all food commodities but in inadequate amounts. PDS, Public Distribution System; HH, Households; HOH, Head of the household; FADI, Food Accessed Diversity Index

**Table 3:** Frequency of food group consumption at household level (*n* = 120) during monsoon season, in Sauria Paharia community of Jharkhand, India.

Food group	Frequency of consumption*, n (%)					
	Daily (2 or more times) Daily (1 time) 3−6 days a week 1−2 days a week Once or twice a month Never					
Cereals and millets	**94 (78.3)**	17 (14.2)	8 (6.7)	1 (0.8)	−	−
Pulses	6(5)	10 (8.3)	30 (25)	**63 (52.5)**	10 (8.3)	1 (0.8)
Green leafy vegetables	3 (2.5)	10 (8.3)	24 (20)	**56 (46.7)**	26 (21.7)	1 (0.8)
Other vegetables	**98 (81.7)**	19 (15.8)	3 (2.5)	−	−	−
Roots and tubers	**106 (88.3)**	12 (10)	2 (1.7)	−	−	−
Fruits	24 (20)	29 (24.2)	**30 (25)**	24 (20)	10 (8.3)	3 (2.5)
Milk and milk products	1 (0.8)	4 (3.3)	7 (5.8)	8 (6.7)	21 (17.5)	**79 (65.9)**
Meat, fish and poultry	4 (3.3)	1 (0.8)	19 (15.8)	**65 (54.4)**	30 (25)	1 (0.8)
Mushrooms	1 (0.8)	−	9 (7.5)	40 (33.3)	**54 (45)**	16 (13.3)
Oils and fats	**106 (88.3)**	10 (8.3)	1 (0.8)	−	−	3 (2.5)
Sugar	24 (20)	**38(31.7)**	29 (24.2)	13 (10.8)	7 (5.8)	9 (7.5)
Market procured packaged and freshly prepared foods	21 (17.5)	**38 (31.7)**	31 (25.8)	30 (25)	−	−

Figures in bold indicate most frequent consumption.

* Some food frequency categories have been merged for easy readability

## Data Availability

The raw data supporting the conclusions of this article will be made available by the authors, without undue reservation.

## Footnotes

1 “Smallholder subsistence farmers” here refers to local food producers who own small, marginal lands (< 10 hectares), often employ family labor and mainly use the farm produce for household consumption. This category also includes people who depend on sale of livestock and livestock products, and are engaged in hunting and foraging foods from forests and water bodies for food and sustenance (Morton, 2007).
